# Current Progress in Peritoneal Dialysis: A Narrative Review of Progress in Peritoneal Dialysis Fluid

**DOI:** 10.3390/life15020279

**Published:** 2025-02-11

**Authors:** Koji Hashimoto, Yuji Kamijo

**Affiliations:** Department of Nephrology, Shinshu University School of Medicine, 3-1-1, Asahi, Matsumoto 390-8621, Nagano, Japan

**Keywords:** peritoneal dialysis, biocompatibility, glucose degradation products, encapsulating peritoneal sclerosis, neutral pH dialysate, icodextrin, lactate, bicarbonate

## Abstract

Peritoneal dialysis (PD) is a renal replacement therapy that removes solutes, electrolytes, and water via the infusion of dialysis fluid into the peritoneal cavity. However, the non-physiological composition of conventional PD fluids can cause peritoneal injury, leading to complications such as peritoneal fibrosis and encapsulating peritoneal sclerosis. This review highlights recent advancements in PD fluid formulations aimed at improving biocompatibility and reducing peritoneal damage. While glucose-based solutions remain the standard because of their affordability, their glucose degradation products (GDPs) and advanced glycation end-products significantly contribute to peritoneal fibrosis. Innovations, such as neutral pH and low-GDP solutions, have been developed to counter these effects, enhancing peritoneal integrity and preserving residual renal function. Alternative osmotic agents, such as icodextrin, offer superior ultrafiltration. Advancements in buffer formulations, including bicarbonate-based and bicarbonate/lactate combinations, have further enhanced the biocompatibility of PD fluids. Despite these progressions, challenges persist. Therefore, future research should prioritize patient-specific PD solutions to optimize long-term outcomes and minimize adverse effects.

## 1. Introduction

Peritoneal dialysis (PD) is a renal replacement therapy that removes solutes, such as uremic toxins and excess electrolytes, as well as water, from the blood by storing dialysis fluid in the abdominal cavity. For effective treatment, the fluid must remain in the abdominal cavity during the dialysis process. Therefore, to facilitate solute and water removal, the composition of dialysis fluid must differ from that of blood, resulting in a non-physiological formulation.

Dialysis fluids may contain osmotic agents to promote water removal, have low sodium and potassium levels to facilitate electrolyte clearance, or include buffers to regulate pH. The most common osmotic agent is glucose; however, glucose undergoes degradation to form glucose degradation products (GDPs) before being administered. Prolonged exposure to such non-physiological solutions can induce structural changes in the peritoneum. Long-term PD increases peritoneal permeability; consequently, the ability to remove water through osmotic agents decreases. As a result, the proportion of patients undergoing long-term PD experience fluid overload is large, and fluid overload is typically the cause of PD discontinuation [1]. In addition, long-term PD can result in encapsulating peritoneal sclerosis (EPS), a severe complication that limits the viability of this therapy.

To mitigate peritoneal damage and address challenges faced by patients undergoing PD, numerous innovations in dialysis solutions have been developed. This review aimed to outline the advancements in PD fluid formulations and their clinical implications.

## 2. Methods

To prepare this narrative review, an initial search was conducted on PubMed on 8 October 2024, using the following query: “peritoneal dialysis” [tiab] AND (“dialysate” [tiab] OR “dialysis fluid” [tiab] OR “dialysis solution” [tiab]) NOT “hemodialysis” [tiab] AND (“review” [Publication Type] OR “review literature as topic” [MeSH Terms] OR “review” [All Fields]). This search provided a foundation of existing knowledge.

Subsequently, from 28 to 30 November 2024, the term “review” was excluded from the original search strategy, and additional terms, including “neutral pH”, “icodextrin”, “sodium”, “calcium”, and “buffer”, representing critical components of PD fluid, were incorporated. Relevant literature was subsequently identified and compiled. We also searched PubMed and the Cochrane Central Register of Controlled Trials to identify relevant clinical studies on PD fluid. Two authors assessed the suitability of the retrieved articles based on the titles and abstracts, and this review was prepared according to the list of selected articles.

The integrated body of collected literature was utilized in the preparation of this review article, which elucidates the evolution of PD fluid.

## 3. Functions Required of PD Fluid

The functions required from an ideal PD fluid are as follows [2]:

- Consistent and efficient water removal;
- Minimal damage to the peritoneal membrane by osmotic agents;
- Stable solute clearance;
- Maintenance of acid–base equilibrium and electrolyte balance under all conditions;
- Inhibition of bacterial proliferation within the dialysis fluid;
- Absence of harmful or toxic substances.

Currently, ideal PD fluids do not exist. However, clinically available PD fluids are selected based on medical requirements or economic considerations. The components of PD fluid can be categorized into osmotic substances, buffers, and electrolytes [3]. These elements have been combined into various formulations to create functional PD solutions. Table 1 outlines the compositions of commercially available PD fluids.

## 4. Osmotic Substances

In PD, osmotic pressure is used to remove water from the dialysis fluid stored in the abdominal cavity through the blood vessels within the peritoneum. The osmotic substances used include glucose, icodextrin, and amino acids.

### 4.1. Glucose-Based Dialysis Fluid

Glucose is the most widely used osmotic substance due to its low cost, availability, and presence in the human body. Glucose-based dialysates are available at various concentrations (1.5%, 2.5%, and 4.25%), and the osmotic pressure can be set over a wide range (344–500 mOsm/L) [4]. The high osmotic gradient facilitates effective fluid removal during the intraperitoneal dwell. However, the ultrafiltration efficiency gradually declines over time when the dialysate is retained in the abdominal cavity, primarily because of dilution from transperitoneal water movement and glucose absorption across the peritoneal membrane. Using a dialysate with high osmotic pressure allows more water to be removed. While higher glucose concentrations enhance water removal, they also accelerate peritoneal mesothelial cell turnover and induce morphological alterations [5]. Recent reports have shown that glucose metabolism in the peritoneum contributes to peritoneal fibrosis via pseudohypoxia [6].

Advanced glycation end-products (AGEs) are derived from glucose and are highly toxic. Honda et al. reported that the accumulation of AGEs in the peritoneum is correlated with peritoneal fibrosis, microvascular sclerosis, and ultrafiltration failure [7]. AGE formation is driven by GDPs generated during the thermal sterilization of glucose-containing solutions. Exposure to GDPs leads to peritoneal mesothelial and vascular endothelial cell damage [8]. The resulting vasculopathy is a finding indicative of peritoneal deterioration along with peritoneal fibrosis and angiogenesis [9]. However, the production of GDPs is unavoidable during the sterilization of the dialysis fluid. Therefore, creating an acidic environment to reduce GDP production is necessary. For this reason, the pH of conventional dialysate was set to an acidic pH of 4.5–5.5, and this was known as acidic dialysate. In addition to the acidic conditions, the presence of GDPs and accumulation of AGEs produced from GDPs damaged the peritoneum, caused ultrafiltration failure, and was a risk factor for EPS [10]. In the development of PD fluids, reducing GDPs and peritoneal damage is crucial.

### 4.2. Glucose-Based Dialysis Fluid: The Era of Neutralization

A dual-chamber system was introduced in the 2000s to reduce peritoneal damage. This system separates an acidic glucose solution from a neutral electrolyte solution, which is mixed immediately before use. The resulting mixed dialysis fluid is neutralized, and the production of GDPs before mixing decreases. Compared to acidic dialysis fluid, neutral pH and lower levels of GDPs reduce peritoneal damage and improve the prognosis of patients undergoing PD.

#### 4.2.1. Effects on Peritoneal Function and EPS

In the era of acidic dialysates, peritoneal permeability increases, and ultrafiltration efficiency decreases over time as PD continues [11]. Elphick et al. reported that, with acidic dialysate, solute transport across the peritoneum exhibited a continuous increase over time, whereas this increase plateaued after 2 years with neutral dialysate [12]. Patients experiencing peritonitis who used acidic dialysates exhibited heightened peritoneal permeability following infection episodes, a phenomenon not observed with neutral dialysate [12]. Peritoneal permeability also increases in patients with EPS [13], and neutral dialysis fluid may have a less harmful effect on the peritoneum than acidic dialysis fluid, potentially suppressing the development of EPS.

In Japan, from 1999 to 2003, when acidic dialysate was used, EPS incidence and mortality rates were reported to be 2.5% and 37.5%, respectively [14]. In contrast, from 2008 to 2012, following the widespread adoption of neutral dialysates, the incidence decreased to 1.0%, with a mortality rate of 35.7% [1]. Histological findings in the peritoneum have shown that the use of neutral dialysis fluid reduces peritoneal damage by decreasing AGE accumulation and inhibiting peritoneal fibrosis [15]. Laparoscopic observation of the abdominal cavity also revealed that prolonged use of acidic fluid caused severe damage to the intestinal visceral peritoneum, resulting in a yellowish, leather-like opacity that obscured the vascular network. In contrast, prolonged use of neutral dialysate led to only mild opacification of the peritoneal membrane, allowing clear visualization of vascular structures [15]. Therefore, the use of neutral dialysis fluid reduced peritoneal damage and suppressed the onset of EPS.

#### 4.2.2. Effect on Residual Renal Function

AGEs contribute to renal damage through pro-inflammatory and pro-oxidative processes [16]. Animal studies have demonstrated elevated AGE levels in rats injected with PD fluid containing high levels of GDPs, leading to morphological changes in the kidneys similar to those observed in diabetic nephropathy [17]. Neutral dialysis fluid, characterized by lower GDP levels, is hypothesized to better preserve residual renal function. Therefore, systematic reviews have shown that neutral dialysates significantly outperform acidic dialysates in maintaining residual renal function [18].

#### 4.2.3. Remaining Issue

Many reports have discussed the effectiveness of neutralized glucose-based dialysis fluid; however, reports caution against peritoneal damage even with neutralized dialysis fluid. Early peritoneal inflammation, fibroblast activation, epithelial–mesenchymal transition, and significant angiogenesis have been reported to be observed after initiating PD using neutral low-GDP dialysate in children [19]. This suggests that, even with the use of neutral low-GDP dialysate, some peritoneal damage is unavoidable due to the use of glucose. Many studies showing the usefulness of neutral low-GDP dialysate have relatively small numbers of cases in intervention trials and short observation periods of up to 24 months, making them insufficient for using the EPS incidence rate as an outcome. From these perspectives, some skepticism remains regarding the biocompatibility of neutral low-GDP dialysate [20]. The development of dialysate with reduced glucose loading is a future issue.

### 4.3. Icodextrin

Icodextrin, a glucose polymer with a molecular weight of 16,800 Da, has been used since the 2000s. It is absorbed more slowly than glucose through the peritoneum and facilitates water removal via colloid osmotic pressure [21]. Icodextrin is gradually absorbed through the lymphatic vessels and metabolized into oligosaccharides and maltose by α-amylase. Tissue maltase finally converts these metabolites into glucose in the cells [22]. Owing to its slow absorption and sustained colloidal osmotic pressure gradient, the ultrafiltration effect persists even with long-term intraperitoneal retention. Additionally, icodextrin has minimal impact on blood glucose levels due to its low glucose load and the production of small GDPs.

A systematic review comparing icodextrin- and glucose-based dialysates confirmed that icodextrin significantly reduces fluid overload episodes and enhances ultrafiltration [18,23]. Therefore, current guidelines recommend its once-daily use for patients experiencing fluid overload with regular glucose-based dialysates [24,25]. Moreover, the European Best Practice Working Group recommends the use of icodextrin in cases of high transporter activity, where water removal is <400 mL after 4 h of storage in 4.25% glucose solution [26]. In Japan, neutralized icodextrin dialysis fluid was released in 2014, which produces fewer GDPs than acidic icodextrin fluid [27] and suppresses morphological changes in mesothelial cells [28]. Although neutralized icodextrin is expected to be effective, clinical data and evidence regarding its efficacy are limited.

Icodextrin reduces glucose load and is useful for fluid management; however, some complications, such as the increase in blood maltose, should be noted. One concern is that, when using a blood glucose analyzer that employs glucose dehydrogenase, the measurement results are affected by metabolites, such as maltose, which are present in the blood other than glucose, making the actual blood glucose level appear higher than it is [29,30]. For patients with diabetes who self-monitor blood glucose, verifying the type of blood glucose analyzer and whether the sensor values are correct is necessary. Another factor is that blood maltose is eventually metabolized into glucose. In PD, high maltose levels can persist for long periods, making its effects on blood glucose levels and body fat unignorable.

### 4.4. Amino Acids

Patients undergoing PD have peritoneal protein loss of approximately 5 g per day [31], which puts them at risk of malnutrition. Therefore, the idea of adding amino acids to the dialysis fluid was conceived, and an amino acid dialysis fluid was developed. In the 1990s, amino acid dialysis fluids, which could be used in some countries, were produced. The amino acid dialysate contains 1.1% amino acids and has an osmotic pressure equivalent to that of 1.5% glucose dialysate [32]. Specifically, the addition of amino acid dialysate improves protein synthesis in patients undergoing PD [33]. However, no significant differences were observed in albumin levels or anthropometric measurements between patients using amino acid dialysate and those using glucose dialysate [34]. Therefore, selecting appropriate cases for use is crucial since the use of amino acid dialysate can cause an increase in urea nitrogen and metabolic acidosis through an increase in nitrogen load. Table 2 summarizes the characteristics of the osmotic substances.

## 5. Buffer

As patients with renal failure present with metabolic acidosis, the dialysate contains a buffer to improve acidosis. To date, the buffers used include acetate, lactate, and bicarbonate.

### 5.1. Acetate

In the early stages of PD, acetate was used as the buffer. Dialysis fluid containing acetate has a stronger antibacterial effect than that containing lactate [35]. However, acetate-containing dialysis fluid takes longer to be metabolized in the abdominal cavity than lactate-containing dialysis fluid, and the time taken for the abdominal cavity to become acidic is longer. This can cause abdominal pain and damage to the peritoneum when the fluid is injected [36]. Therefore, acetate-containing dialysis fluid is no longer used due to its harmful effects on the peritoneum.

### 5.2. Lactate

Lactate is metabolized in the body and becomes a source of bicarbonate, which alleviates acidosis. It is present in the PD fluid at a concentration of 35–40 mEq/L. The load of lactate absorbed in PD is significantly lower than the rate of lactate production in healthy people (15–30 mEq/kg/day); therefore, no reports have revealed elevated serum lactate levels in patients undergoing PD [37]. The metabolic pathway is clear and widely used as a buffer in PD fluids. However, lactate-containing dialysis fluid is harmful to peritoneal mesothelial cells [38,39] and affects peritoneal angiogenesis and fibrosis [40].

### 5.3. Bicarbonate

Initially, creating a dialysate containing bicarbonate was impossible because adding bicarbonate to the PD fluid would cause calcium and magnesium carbonates to precipitate. However, the use of a dual-chamber system enables the separation of the chamber containing bicarbonate from that containing electrolytes, facilitating the use of a dialysate containing bicarbonate.

Bicarbonate dialysate is more biocompatible than lactate-based dialysate and has fewer adverse effects on peritoneal mesothelial cells [41]. It also produces fewer GDPs than lactate-based dialysates [42]. Pure bicarbonate-based dialysate and dialysate containing a mixture of bicarbonate and lactate are currently available. However, dialysate containing only bicarbonate may cause intracellular acidosis due to an increase in dialysate CO_2_ concentration [43]. The infusion pain is reduced with bicarbonate/lactate dialysate compared to pure bicarbonate dialysate [44], and bicarbonate/lactate dialysate is generally more suitable [43]. Notably, the target bicarbonate level differs depending on the type of dialysate. A study compared patients using PD fluid containing 40 mmol/L lactate with those who continued using the same fluid or switched to dialysis fluid containing 25 mmol/L bicarbonate and 10 mmol/L lactate. The group that continued using a dialysis fluid with 40 mmol/L lactate exhibited abnormally high blood bicarbonate levels, whereas the group that switched to 25 mmol/L bicarbonate and 10 mmol/L lactate-containing dialysis fluid experienced a reduction in blood bicarbonate levels [45]. International observational studies have shown that a high bicarbonate concentration in the dialysis fluid for patients undergoing hemodialysis is a risk factor for death via metabolic alkalosis after dialysis [46]. Since the acid load situation changes depending on the patient’s body size, diet, activity level, and other factors, selecting a dialysis fluid that considers the target value of the buffer set for each PD fluid is necessary. Table 3 summarizes the characteristics of the buffer.

## 6. Electrolytes

### 6.1. Sodium

Patients undergoing PD are prone to hypertension and fluid overload due to residual renal function decline [47]. To control fluid overload and remove sodium, the sodium concentration of the PD fluid is set to 132–135 mEq/L, which is lower than the serum sodium level. This allows continuous ambulatory PD to remove approximately 90 mEq of sodium or water from the body [43].

Studies have also explored low-sodium dialysates, which have even lower sodium concentrations. Low-sodium dialysate reportedly increased peritoneal sodium removal and reduced body weight and mean blood pressure [48,49]. However, recent randomized controlled trials have failed to show any benefits of low-sodium dialysates over conventional dialysates. Additionally, their use has been associated with more hypotensive episodes [50]. The evaluation of low-sodium dialysates has not yet been established.

### 6.2. Potassium

Less potassium is removed by PD because the amount of dialysis fluid that can be used daily is smaller for PD than for hemodialysis. Therefore, PD fluid does not contain potassium and can remove 30–40 mEq/day of potassium. During hemodialysis, 60 mEq/L of potassium was removed per treatment [51]. The efficiencies of potassium removal during hemodialysis and PD were equivalent per week. In contrast, hypokalemia is more likely to occur in patients undergoing PD than in those receiving hemodialysis [52]. Hypokalemia in patients undergoing PD is associated with total mortality, cardiovascular mortality, and the development of PD-related peritonitis [53]. Therefore, physicians must prevent hypokalemia development, and potassium supplementation is required when necessary.

### 6.3. Calcium

As patients with renal failure tend to have low calcium levels, dialysis fluids with a calcium concentration of 3.5–4.0 mEq/L were developed to provide calcium supplementation. However, because the use of calcium-containing phosphate binders and vitamin D preparations can cause hypercalcemia, dialysis fluids with low calcium concentrations have been developed. Therefore, dialysis solutions with a calcium concentration of 2.5–4.0 mEq/L are currently available. Low-calcium dialysate has enabled the use of calcium-containing phosphate binders and vitamin D preparations at higher doses; however, parathyroid hormone levels have been reported to increase in patients using low-calcium dialysate [54]. A study comparing low-calcium dialysate (1.25 mEq/L) with high-calcium dialysate (1.75 mEq/L) also suggested that high-calcium dialysate is associated with vascular calcification [55]. Recently, calciprotein particles (CPPs) in the serum of patients with renal disease and those with chronic inflammation have been associated with driving sterile inflammation and extraosseous mineral deposition. CPP formation in the body was promoted by PD fluid with a calcium concentration of 1.75 mmol/L compared to that with a calcium concentration of 1.25 mmol/L. The osmotic pressure regulator, pH of the fluid, and glucose concentration did not affect the formation of CPPs [56]. These findings suggest that dialysis fluid with a high calcium concentration affects vascular calcification via the formation of CPPs. In contrast, using low-calcium dialysate is associated with the need for more phosphate binders and antihypertensive drugs than when using high-calcium dialysate, and no significant difference in arterial calcification was observed [57]. Recently, the frequency of the use of calcium-lowering drugs, such as calcimimetics and bone resorption inhibitors, for osteoporosis treatment has also increased. Therefore, selecting a dialysate that matches the calcium status of an individual is crucial.

### 6.4. Magnesium

The magnesium concentration of PD fluid is set at 0.5–1.5 mEq/L. However, the use of 1.5 mEq/L dialysis fluid increases the frequency of hypermagnesemia; therefore, because hypermagnesemia increases osteopenia, the use of low-magnesium dialysis fluid has become widespread [21]. In the case of normal kidneys, 2–8 mEq/day of magnesium is excreted. When using a PD fluid with a magnesium concentration of 0.5 mEq/L, 1.0–2.0 mEq of magnesium is removed via the peritoneum [43]. Compared to the kidney, less magnesium is removed via PD; however, hypomagnesemia is a risk factor for cardiovascular and non-vascular mortality due to infection in patients undergoing PD [58]. Hypomagnesemia must be considered when using low-magnesium dialysates.

## 7. Innovative Advances in PD Fluid

PD fluid comprises the abovementioned components; however, even neutralized, low-GDP PD fluid has been shown to induce structural alterations in the peritoneum [19]. Efforts are underway to develop novel dialysis solutions aimed at enhancing the outcomes of PD. A study has been performed on the use of L-carnitine as an osmotic agent to reduce glucose load [59]. Recently, a dialysis fluid containing xylitol and L-carnitine has been shown to reduce the glucose content and protect the viability of peritoneal mesothelial and vascular endothelial cells in vitro [60]. Moreover, it does not appear to exacerbate fibrosis, inflammation, or angiogenesis compared to conventional glucose-based solutions. Clinical trials involving patients undergoing PD have confirmed its safety for administration over 4 weeks, with stable dialysis efficiency and fluid management parameters [61]. A phase III randomized controlled study is currently being planned to investigate the efficacy and safety of xylitol and L-carnitine dialysis fluid [62]. By reducing the glucose load, this innovative solution holds promise for improved biocompatibility and clinical utility.

Additionally, the incorporation of alanyl-glutamine (AlaGln) into PD fluid has been reported to restore cellular stress responses and enhance the immune functionality of peritoneal white blood cells. A randomized controlled trial revealed that AlaGln-supplemented PD fluid significantly improved biomarkers associated with peritoneal membrane integrity, immune competence, and systemic inflammation compared to unsupplemented neutral pH, low-GDP solutions [63]. No safety concerns were identified. Furthermore, peritonitis was observed in patients using standard PD fluids without AlaGln but not in those receiving AlaGln-enriched solutions, suggesting its potential role in preventing peritonitis. However, no reports exist of clinical studies involving large numbers of patients using AlaGln-enriched dialysis fluid, and the evidence for its efficacy has not been established.

The protective effects of molecular hydrogen (H_2_) added to PD fluid are also under investigation. Studies indicate that H_2_-enriched solutions mitigate peritoneal damage and may contribute to maintaining peritoneal integrity [64]. Clinical trials reported no adverse effects, with some patients exhibiting significant improvements in peritoneal mesothelial cell markers in the effluent, suggesting a role for H_2_ in promoting mesothelial cell regeneration [65]. The stability of H_2_ in PD fluid is an issue to be resolved. Additionally, only a limited number of reports on H_2_-enriched solutions are currently available, and further investigation is needed to determine their effectiveness.

In addition to the above, attempts have also been made to optimize the infusion program in automated PD as a way of reducing glucose load [66]. In this study, optimization improved ultrafiltration per 1 g of glucose absorption and Kt/V. However, it has not yet been fully evaluated since this is only a pilot study. In addition to the evolution of dialysis fluid, it is believed that the manner in which it is used could represent an effective direction for future PD.

These advances underscore the potential of novel PD fluids to enhance biocompatibility, reduce complications such as fluid overload due to insufficient water removal, and improve the outcomes of PD.

## 8. Limitations

First, this study uses a narrative review approach and is not based on a strict review protocol such as a scoping or systematic review. Second, a high risk of selection bias exists since the literature was selected based on the authors’ subjective judgment. Third, many of the papers used in this review were based on small numbers of cases and facilities, and a high risk of potential bias exists. Finally, most of the research on PD is of this type, highlighting the need for well-controlled, large-scale clinical studies.

## 9. Conclusions

The evolution of dialysis fluids, with a focus on the individual components of PD fluid, was evaluated. Significant efforts in PD have been dedicated to mitigating the detrimental effects of non-physiological dialysis solutions on the peritoneum to preserve peritoneal functionality and prevent EPS. Although the biocompatibility of PD solutions has improved significantly over time, it remains suboptimal. Moreover, evolving clinical demands driven by the increasing number of patients undergoing dialysis, an aging population, and the advent of novel pharmacological agents necessitate continuous innovation. Sustained advancements in PD are imperative to effectively address these challenges. This review also confirmed a lack of high-quality evidence in PD. Therefore, high-quality controlled studies are needed to determine whether existing approaches to improving biocompatibility are truly effective or to demonstrate the efficacy of new drugs.

## Figures and Tables

**Table 1:** Currently available peritoneal dialysis fluid.

Contents	Range
Osmotic substances	
Glucose	1.36–4.25%
Icodextrin	7.5%
Amino acid	1.1%
Electrolytes	
Sodium	132–135 mEq/L
Potassium	0 mEq/L
Calcium	2.5–4.0 mEq/L
Magnesium	0.5–1.5 mEq/L
Buffer	
Lactate	35–40 mEq/L
Lactate + Bicarbonate	10–15 + 25 mEq/L
Bicarbonate	34 mEq/L
pH	4.5–7.8

**Table 2:** Summary of the characteristics of osmotic substances.

Contents	Advantage	Disadvantage
Glucose–acidic	inexpensive	glucose load high GDPs
Glucose–neutralized	inexpensive lower GDPs	glucose load dual-chamber system operation
Icodextrin	lower glucose load increase fluid removal	accumulation of maltose pseudohyperglycemia
Amino acid	lower glucose load protein supplement	elevated blood nitrogen acid load

GDPs, glucose degradation products.

**Table 3:** Summary of the characteristics of the buffer.

Contents	Advantage	Disadvantage
Acetate	strong antibacterial ability	infusion pain high risk of peritoneal damage
Lactate	high stability	risk of peritoneal damage
Bicarbonate	high biocompatibility low GDPs production	high CO_2_ concentration in dialysate infusion pain
Bicarbonate/lactate	High biocompatibility	high CO_2_ concentration in dialysate

GDPs, glucose degradation products.

## Data Availability

No new data were created or analyzed in this study. Data sharing is not applicable to this article.

## Abbreviations

The following abbreviations are used in this manuscript:

PD	Peritoneal dialysis
EPS	Encapsulating peritoneal sclerosis
GDP	Glucose degradation product
AGE	Advanced glycation end-product
AlaGln	Alanyl-glutamine
H2	Hydrogen
